# Using the Fusion Proximal Area Method and Gravity Method to Identify Areas with Physician Shortages

**DOI:** 10.1371/journal.pone.0163504

**Published:** 2016-10-03

**Authors:** Xuechen Xiong, Chao Jin, Haile Chen, Li Luo

**Affiliations:** Collaborative Innovation Center of Health Risks Governance, School of Public Health, Fudan University, Shanghai, 200433, China; Peking University, CHINA

## Abstract

**Objectives:**

This paper presents a geographic information system (GIS)-based proximal area method and gravity method for identifying areas with physician shortages. The innovation of this paper is that it uses the appropriate methods to discover each type of health resource and then integrates all these methods to assess spatial access to health resources using population distribution data. In this way, spatial access to health resources for an entire city can be visualized in one neat package, which can help health policy makers quickly comprehend realistic distributions of health resources at a macro level.

**Methods:**

First, classify health resources according to the trade areas of the patients they serve. Second, apply an appropriate method to each different type of health resource to measure spatial access to those resources. Third, integrate all types of access using population distribution data.

**Results:**

In case study of Shanghai with the fusion method, areas with physician shortages are located primarily in suburban districts, especially in district junction areas. The result suggests that the government of Shanghai should pay more attention to these areas by investing in new or relocating existing health resources.

**Conclusion:**

The fusion method is demonstrated to be more accurate and practicable than using a single method to assess spatial access to health resources.

## Introduction

Access to healthcare varies from place to place because healthcare suppliers and healthcare demands are distributed unevenly. Healthcare resources are always scarce, while health care demands increase rapidly. Therefore, it is crucial to ensure that the provision of health resources is equitable and that access to health resources is adequate. To accomplish these tasks, we need effective planning to allocate health resources in ways that best satisfy people’s health demands.

In recent years, various methods including population-to-provider ratios, the minimum distance method, the 2-step floating method, and the gravity method, along with some modifications and extensions of these original methods[1–3] have been proposed to identify underserved or overserved areas by calculating access to health resources and pinpointing regions where service provision should be increased or reduced[4–6].

The simplest method is to calculate the ratio of health care providers to population within a given region. Most such regions are formed by official boundaries such as counties or cities. The U.S. Department of Health and Human Services (DHHS) uses the population-to-physician ratio within a “rational service area” as a basic indicator for defining physician shortage areas[7]. Despite being simple and intuitive, this approach has two obvious weaknesses. On the one hand, a supply-demand ratio reveals the general availability of health resources within a given unit area but cannot account for detailed spatial variation within that unit. On the other hand, this approach ignores cross-regional travel by patients seeking high-end health care[8] because it assumes that all healthcare demands are met by health resources supplied only within the units themselves. For instance, in areas that have been designated as physician shortage areas by the DHHS, the population-to-physician ratio is often calculated at the county level, implying that residents do not visit physicians beyond county borders[7]. To overcome the shortcomings mentioned above, Luo came up with the two-step floating catchment area (2SFCA) method to assess physician shortage areas[8]. The 2SFCA method draws an artificial line between accessible and inaccessible locations as a floating catchment area. The catchment area can be drawn as a circle[9] or as a fixed travel-time range[10]. In this way, the boundary of an administrative unit can be identified by floating catchment areas. However, similar to the supply-demand ratio method, supply and demand can be handled only within a certain range, and all supply and demand instances that occur within the same area range are counted equally—without accounting for differences in travel distance or time. In other words, the 2SFCA identifies an artificial line; resources beyond that line are considered inaccessible, while people inside the area enclosed by the line are considered to have equal access to health resources. The 2SFCA method treats distance (time) impedance as a dichotomous measure; that is, any distance (time) within the threshold is equally accessible and any distance (time) beyond the threshold is equally inaccessible[8]. The biggest shortcoming of the 2SFCA method is that it assumes that access does not diminish with distance within a catchment area[11]. Many studies have attempted to improve it, and most are from healthcare-related applications. These methods can be synthesized together in one framework, which is called “general 2SFCA method” [7]. The gravity model, as one special case of the “general 2SFCA method”, which rates nearby suppliers as more accessible than remote ones, reflects a continuous decay of access based on distance. Even through the gravity-based method is considered to be the best for evaluating spatial accessibility and is deemed more conceptually sound than the 2SFCA method[12], the 2SFCA method is still a better choice in many cases. Criticisms of the gravity model focus mainly on the difficulty of calculating the distance-decay function, which requires more data input. The model requires prior knowledge of the number and locations of healthcare suppliers, the demand locations, the traffic network, and finally, the analysis of travel time between the supply locations and the demand locations[13]. In addition, the friction coefficient β used in the distance decay function must be determined by physician-patient interaction data—and it may be region specific[14].

Considering the respective advantages and disadvantages of the methods mentioned above, choosing which one to use to measure access to health resources in a large area is confusing to health-related policy makers. Obviously, any single method cannot reveal detailed spatial accessibility variations accurately and realistically within a large area.

The purpose of this study is to find a way to integrate the different methods for measuring spatial access to health resources within a large area, where various types of health resources—from primary to high-end—are provided. The key players in all types of health resources are physicians; in this paper, we use Shanghai as a test area for visualizing spatial access to physicians. The method used in this paper can be considered as requiring four steps: (1) visualize the distribution of health resources in the community using the supply-demand ratio method, which is calculated within administrative boundaries of the community; (2) calculate patients versus population in the service area designated by the proximal area method; (3) estimate the friction coefficient β for first-class hospitals, and then visualize spatial access to first-class physicians using the gravity method; (4) integrate the results of steps (1), (2), and (3) with population distribution data to create a visualization of spatial access to physicians in an entire region. Finally, use the visualization to identify areas with physician shortages.

## Background

### Medical care system of China

China organizes parallel three-tier health organizations to deliver healthcare to rural and urban residents. In rural sectors, the tiers consist of village stations, township stations, and county hospitals. In urban sectors, they are street health stations, community health centers, and district hospitals. The most serious illnesses are referred by country hospitals to city or regional hospitals[15]. In this system, hospitals are classified into first-, second-, and third-grade hospitals. A first-grade hospital refers to township stations and community health centers that provide preventive measures and basic health services for local residents. A second-grade hospital refers to county or district hospitals, which provide healthcare for people in nearby areas such as curing common or treating ongoing diseases. Second-grade hospitals usually serve across communities, while third-grade hospitals, also called “first-class hospitals,” serve people from even larger areas, supplying high-end health services and products. Note that in China the government supplies all healthcare resources. Therefore, the government is responsible for ensuring that people’s heath achieves the goal of universal access to health care 2000 [16].

### Data

#### Administrative boundary data

**Fig 1:**
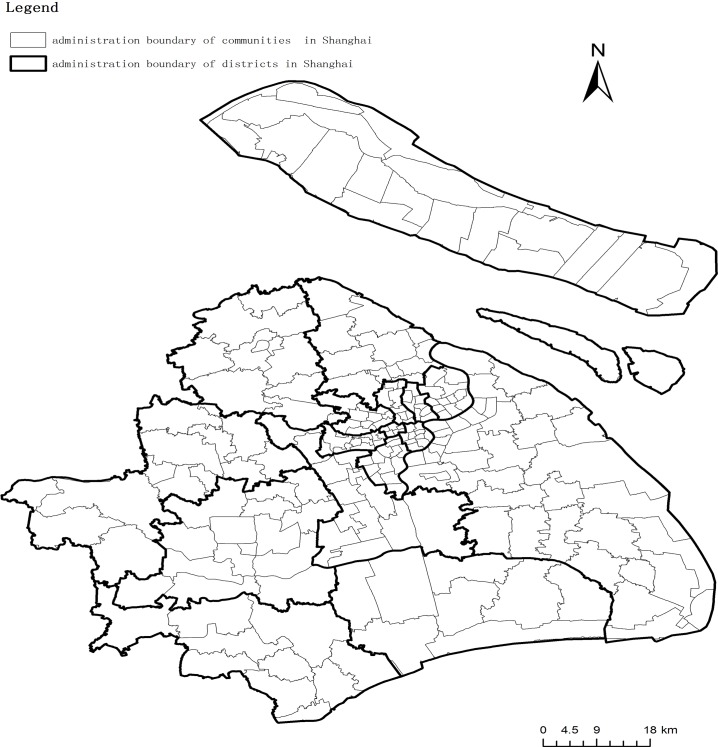
Shanghai’s administration boundary.

Shanghai is currently divided into 16 districts and 1 county (Chongming). Among these districts, Jingan, Xuhui, Yangpu, Huangpu, Hongkou, Zhabei, Putuo, Changning are located in the central city (Fig 1). Specifically, these districts and the county are separated into 212 communities (Fig 1) and 5974 sub-districts (accurate boundaries for the sub-districts are difficult to obtain). The source for this study consisted of administrative boundary data collected by the Urban Planning Bureau of Shanghai in 2011.

#### Population data

**Fig 2:**
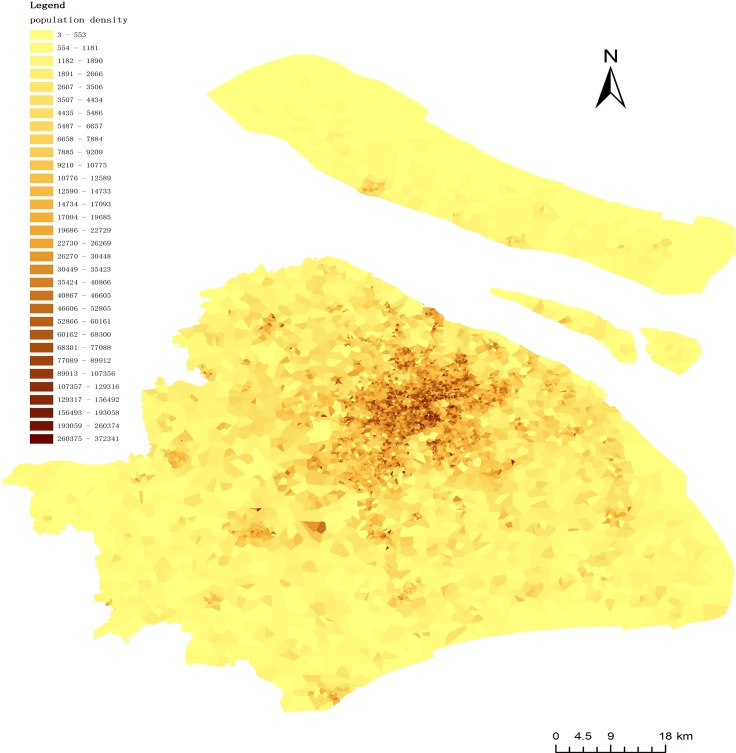
Population density.

Shanghai, a large city in China, has an approximate population of 25,000,000 people living within a 7200 square kilometer area. The addresses of 5974 sub-district committees were used along with population data for each sub-district to simulate the population distribution of Shanghai in ArcGIS 10.0 (Fig 2 shows the population density of Shanghai). This study used population data for 2014 collected by the Public Security Bureau of Shanghai.

#### Physician data

**Fig 3:**
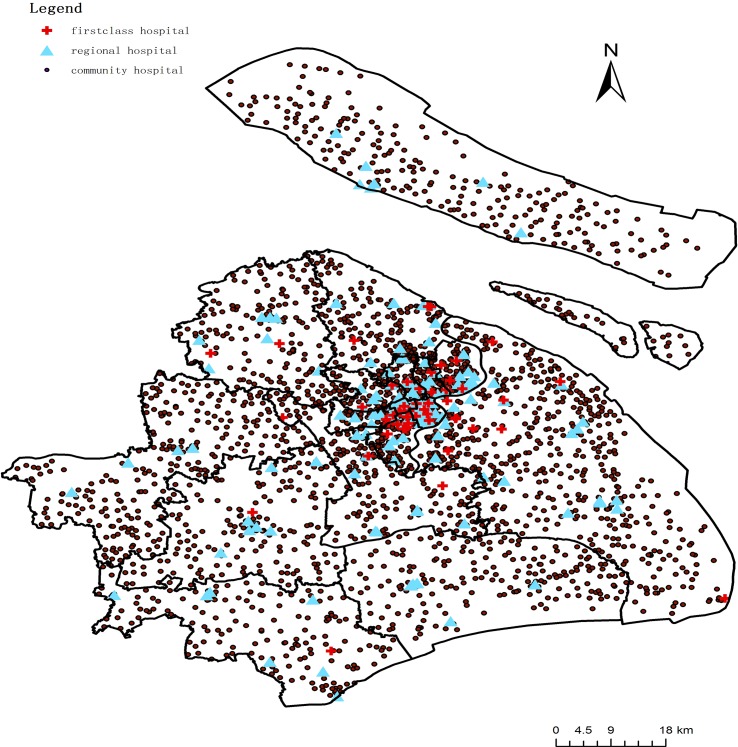
Distribution of health institutions.

Taking into account the hospital grade and the practical influence of each hospital through 2014, Shanghai has 51 first-class hospitals with 22,699 physicians serving people from Eastern China, 145 regional hospitals with 22,610 physicians serving nearby residents, and 2586 community hospitals/sites with 19,402 physicians serving local residents. All these institutions were established and are maintained by government. Private health institutions account for less than 10% and most of these are walk-in clinics. So this paper does not take physicians working in private hospitals into account. All data for health institutions and physicians were collected by the Health Bureau of Shanghai for 2014 (Fig 3).

## Method

### The physician-to-population method for community hospitals

**Fig 4:**
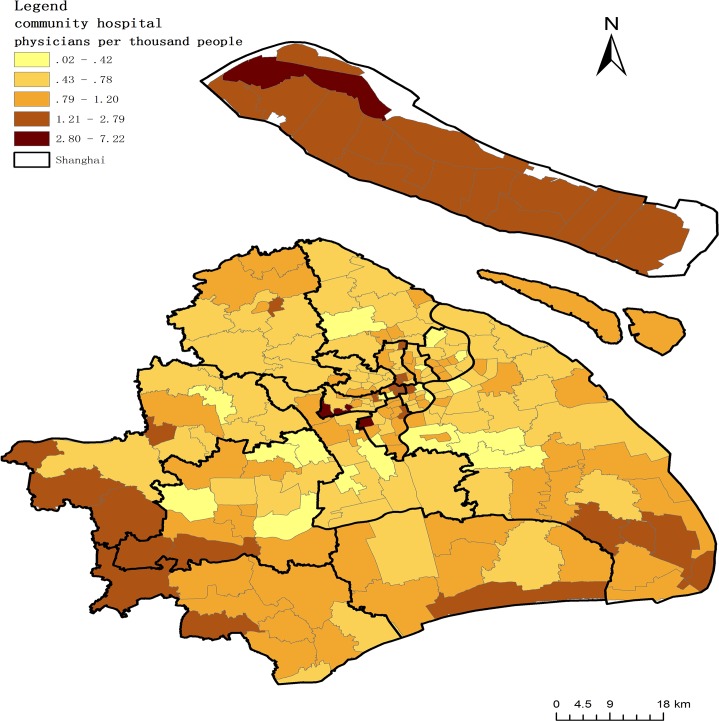
Accessibility to physicians in community hospitals.

There are 2586 community hospitals/sites established in Shanghai. Because community hospitals is intended for local residents, full-time physicians working in community hospitals actually serve any people in the local area. Even though people are free to choose regional hospitals or other first-class hospitals instead, basic health demands are usually satisfied adequately by local community hospitals. In other words, community physicians rarely accept patients from other communities. Therefore, it is straightforward to associate physicians in community hospitals with people who live in the community. Then, access to community physicians can be measured by Formula (1).

$$A_{1}={Ph}_{1}/{Po}_{1}$$ (1)

where *A*_1_ represents the overall access to physicians in each community, *Ph*_1_ is the number of physicians in each community, and *Po*_1_ is the population of each community. All these variables are calculated at the community level. Fig 4 shows the accessibility to physicians in community hospitals.

### The proximal area method for regional hospitals

The service areas of regional hospitals are not restricted to supplying healthcare only within a particular administrative boundary. Therefore, it is not suitable to treat district administrative boundaries as the service area for regional hospitals. The distribution of regional hospitals should be equitable to ensure that everybody can access superior health services conveniently. Because the competence level at each regional hospital is similar, distance is the primary factor for patients seeking heath resources from any regional hospital. So the next task becomes a matter of determining an equitable distribution of regional hospitals by considering distance as a factor. The proximal area method is a simple geographic approach for defining trade areas[7]. It assumes that patients choose the nearest hospital among similar hospitals. This assumption is also found in the classical central place theory[17]. The proximal area method was established using the assumption that distance (or travel time) is the only consideration in people’s consumption choices. Applying this theory to patient choice among regional hospitals, as discussed, travel distance is the only consideration. Thus the service area of a regional hospital is made up of patients who are closer to it than to any other regional hospital. With this service area defined, physicians can be distributed to serve the population within the service area.

**Fig 5:**
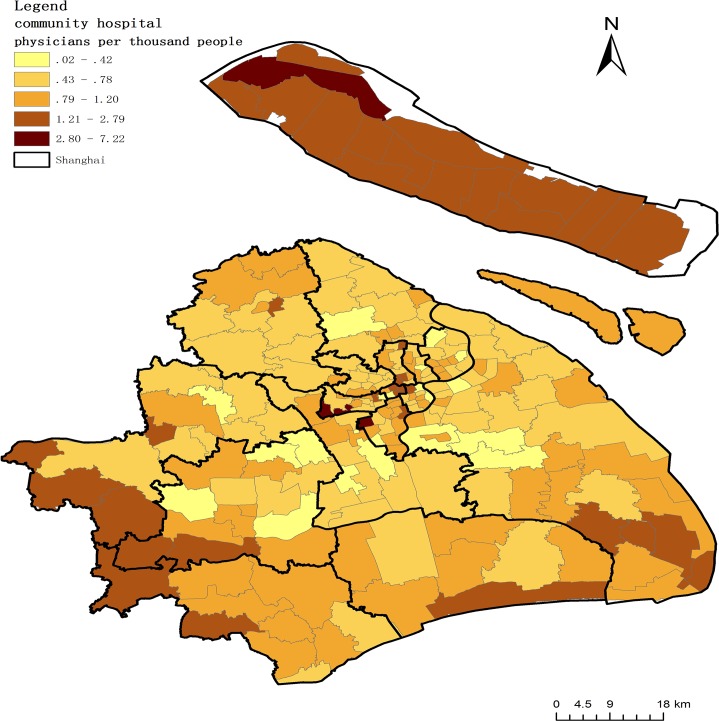
Accessibility to physicians in regional hospitals.

This case study of Shanghai used the locations of 47 typical regional hospitals to generate the Thiessen polygons. These 47 typical regional hospitals have been designated as regional hospitals among all second-grade hospitals in the policy document of Shanghai’s regional health planning (2010–2020) [18]. The access to physicians working in regional hospitals is measured by Formula (2).

$$A_{2}={Ph}_{2}/{Po}_{2}$$ (2)

where *A*_2_ is the access to physicians in regional hospitals, *Ph*_2_ is the number of physicians at regional hospitals in each service area, and *Po*_2_ is the population in each service area. Fig 5 shows the accessibility of physicians in regional hospitals.

### The gravity method for first-class hospitals

The proximal area method describe above works under the assumption that patients will not seek health resources outside their service area. This assumption is conditionally accepted, so the proximal area method is appropriately used for regional hospitals. However, patients may bypass the closest hospital to patronize some other hospital with better health services, better products, or a better reputation. In additional, a hospital in proximity to other hospitals may also attract patients from further away than an isolated hospital because of the aggregation effect. In the case study of Shanghai, apart from the community and regional hospitals, there are 51 first-class hospitals serving both people in Shanghai and throughout eastern China. Therefore, the 22699 physicians in these first-class hospitals not only serve people in the region but also patients from far more remote areas. The problem here is to distribute this type of health resource to the population scientifically, in a manner consistent with the facts. The gravity method can solve this problem.

The gravity model is:

$$A_{i}^{G}=\sum_{j=1}^{n}\frac{S_{j}{d_{ij}}^{-\beta }}{V_{j}},\;where\;V_{j}=\sum_{k=1}^{m}D_{k}d_{kj}^{-\beta }$$

and where $A_{i}^{G}$ is the gravity-based access at location i, S_j_ is the supply capacity at location j, d_ij_ is the distance or travel time between the demand (at location i) and a supply location j, β is the travel friction coefficient, n is the total number of supply locations, and m is the total number of demand locations[19]. V_j_ is measured by its population potential. This model not only considers supply but also considers demand. Essentially, the gravity model is also the ratio of supply to demand, but weighted by travel distance (or time) to a negative power (β). However, there is a problem yet to be solved—how to figure out parameter of β. It’s particularly difficult to define the travel friction coefficient β in the gravity model because β varies from place to place and occurs over time[20]. An earlier study used β = 1.5 and β = 2 to determine the spatial accessibility of health resources[21].

In our case study of Shanghai, there are 51 first-class hospitals serving all of Shanghai, or all of eastern China. Therefore, we use the gravity model to distribute physicians working in these facilities throughout Shanghai. Supply is the number of physicians located in hospitals; demand is based on the population. Now, accessibility to physicians working in first-class hospitals can be calculated by the gravity model. All the calculations can be performed in ArcGIS 10.0.

### Establish the hospitalization probability function model

In this paper, we pick Zhongshan Hospital as a typical first-class hospital to establish a hospitalization probability function model to estimate the parameter of the travel friction coefficient β. It has been validated that the power equation is good for fitting the relationship between distance and hospitalization probability. So the power model of non-linear regression was adopted to fit the curve of probability of hospitalization. In 2014, Zhongshan Hospital served approximately 76144 inpatients. Approximately 51% of the total inpatients traveled from other provinces—were not Shanghai residents.

**Fig 6:**
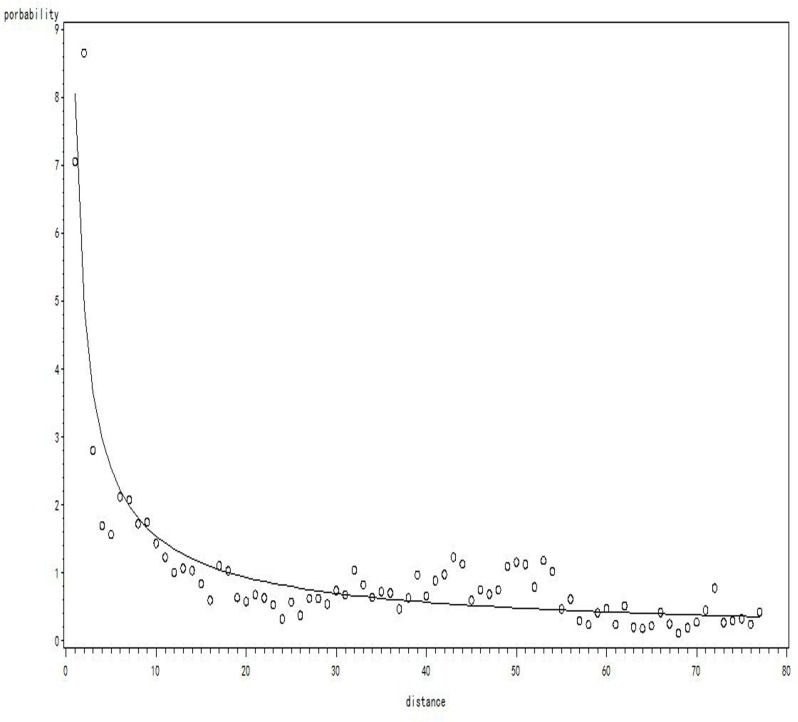
The scatterplot of hospitalization probability and the curve of the probability distribution.

The distances between the location of Zhongshan Hospital and inpatients’ residences is used together with the probability of patients being hospitalized in Zhongshan to fit the power model to calculate the travel friction coefficient β. The following steps briefly introduce the procedure to calculate β: (1) Create a multiple ring buffer zone covering Shanghai with a 1-km ring radius centered on Zhongshan Hospital. (2) Count the number of patients and the total population in each ring buffer to compute the probability of hospitalization. This approach is implemented by utilizing the “SpatialJoin” tool. (3) Adopt the power model of non-linear regression to fit the curve of probability of hospitalization. Fig 6 shows a scatterplot of hospitalization probability and the curve of the probability distribution.

By following these steps, the function model can be established and the parameter β for the distance decay coefficient can be calculated. The result is listed in Table 1.

**Table 1:** The result of hospitalization probability function model.

	α	*β*	P	R^2^
P = α * *D*^*β*^	8.05	-0.7	<0.001	0.87

The power model is:

$$P=\alpha \;*\;D^{\beta }$$

where P is the probability of residents being hospitalized in Zhongshan Hospital, α is a proportionality factor, *D* is the distance between the addresses of patients and the location of Zhongshan Hospital, the unit of “D” is in kilometers, and *β* is the travel friction coefficient.

As a result, the power mode was established,

$$P=8.05\;*\;D^{-0.7}$$

**Fig 7:**
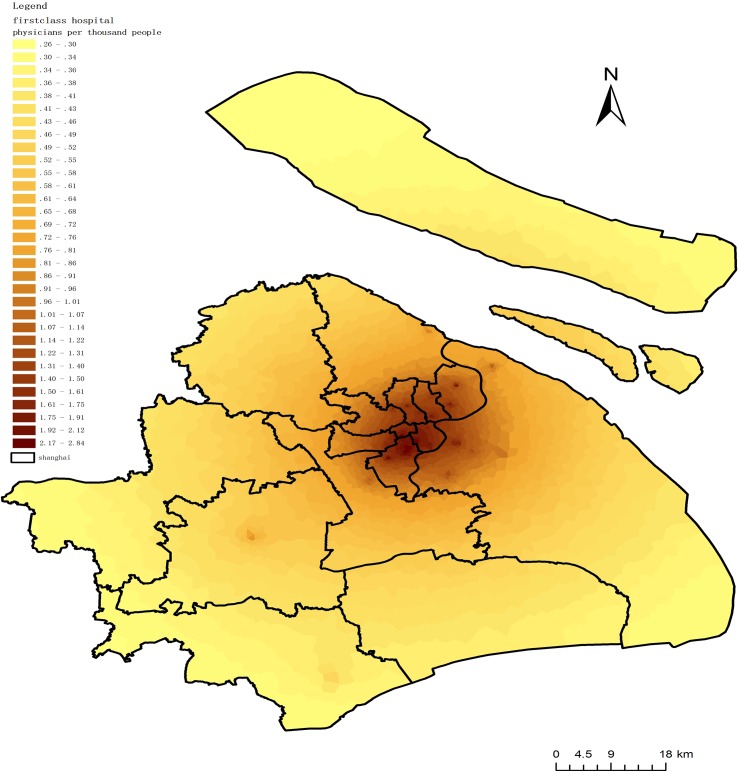
Spatial access to physicians in first-class hospitals.

In this paper *β* = -0.7 is applied in all 51 first-class hospitals to measure physician accessibility to residents based on the principle of gravity model. Fig 7 shows the physician distribution of these 51 first-class hospitals throughout all of Shanghai. All calculations were performed in ArcGIS 10.0 and SAS 9.2.

$$A_{3}=\sum_{j=1}^{n}\frac{S_{j}{d_{ij}}^{-0.7}}{V_{j}},where\;V_{j}=\sum_{k=1}^{m}D_{k}d_{kj}^{-0.7}$$

### Integration

Physicians in Shanghai are classified into three categories according to their service area: those working in community hospitals, those working in regional hospitals, and those working in first-class hospitals. These three types of physicians have been distributed to the appropriate set of residents in the previous section of this paper. Physicians in community hospitals are distributed to residents within the administrative boundary of that community using the physician-to-population method. Physicians in regional hospitals are distributed to residents living in the hospital’s service area as drawn by the proximal area method. Physicians in first-class hospitals are distributed to people in all of Shanghai using the gravity model method. Consequently, we can obtain three types of physician-to-population values: physician-to-population value within community administrative boundaries (A_1_), physician-to-population value within the service areas of regional hospitals (A_2_), and physician-to-population value for the entire area of Shanghai (A_3_).

**Fig 8:**
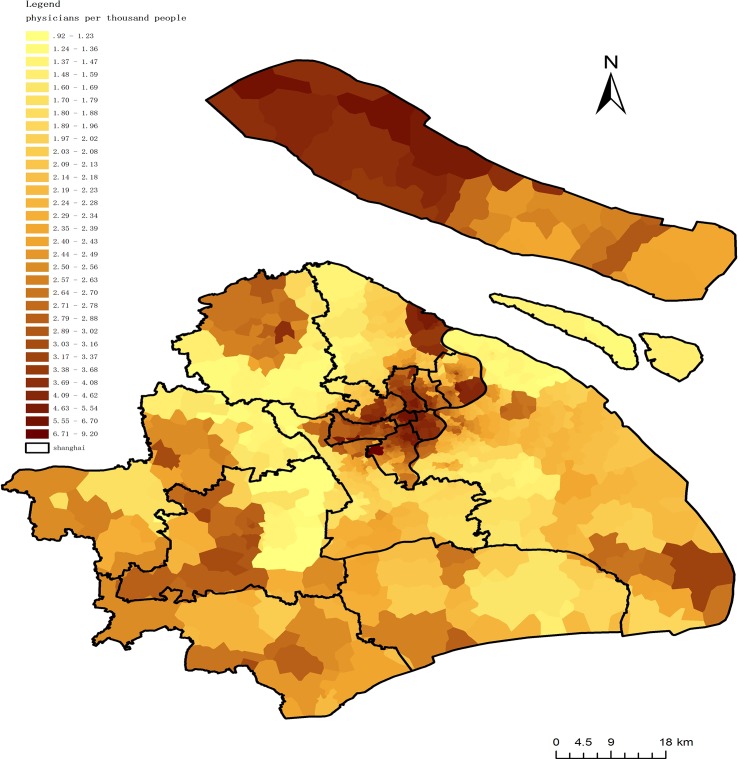
Spatial access to physicians in Shanghai.

To determine people’s access to all physicians, this paper uses the population distribution data around Shanghai as a key link between the fusion proximal area method and the gravity method. The steps of the operation are as follows: (1) collect and establish a point layer of population locations; (2) assignment the physician-to-population ratio for each community (A_1_) to every population point within the community boundary; (3) assignment the physician-to-population ratio of regional hospitals (A_2_) to every population point within the regional hospital service area; (4) assignment the physician-to-population ratio of first-class hospitals (A_3_) to every population point within Shanghai (Step 4 was already performed when calculating accessibility using the gravity model method and Steps 2 and 3 are implemented by utilizing the “SpatialJoin” tool in ArcGIS 10.0); (5) at this point, every population point has three types of physician-to-population ratios representing that point’s accessibility with regard to the three types of physicians; therefore, add the three types of physician-to-population ratios to obtain a total access-to-physician ratio for each population point, (6) create a visualization of the spatial access to all physicians in Shanghai (Fig 8).

## Results

In this article, physicians are classified into three groups according to the area and patients they serve. These groups are: physicians working in community hospitals serving residents who live in the same community, physicians working in regional hospitals serving people who live in nearby areas, and physicians working in first-class hospitals serving people from larger areas. Next, using a simple physician-to-population approach, the availability of community hospitals at the community level is measured using the principle of proximal area method to measure the availability of regional hospitals by calculating physician-to-population values for the region, and the gravity method is used to measure spatial access to high-end medical resources by calculating the distance-decay function. Finally, these methods are integrated to visualize spatial access to health resources with the aid of population distribution data. The results show realistic spatial access to physicians and highlight areas with physician shortages in a case study of Shanghai, China.

**Fig 9:**
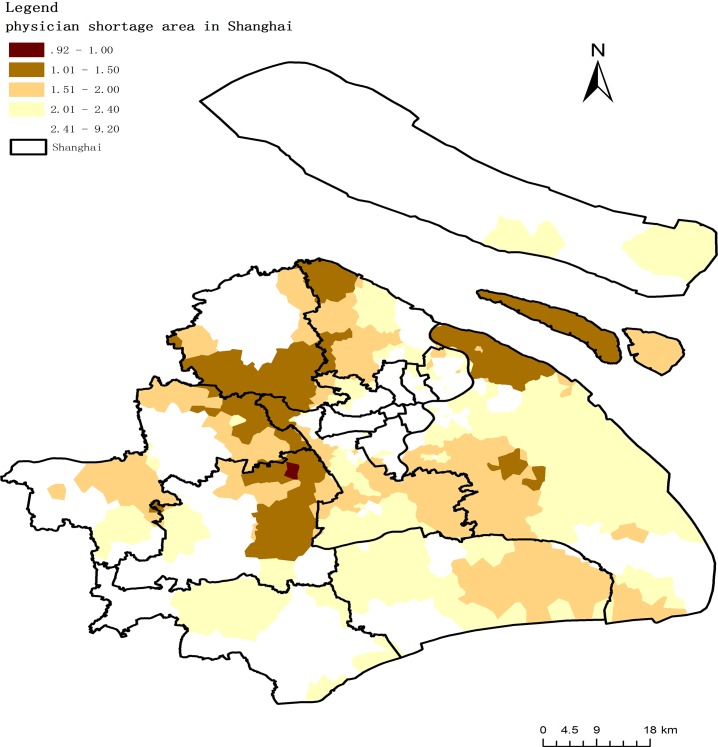
Physician shortage areas in Shanghai.

After calculating the access-to-physician ratio for Shanghai, along with the standard physician-to-patient ratio, areas with physician shortages can be explicitly revealed. According to the policy of Shanghai’s regional health planning (2011–2020), physician to population ratio is going to up to 2.4:1000 in the year of 2020. Fig 9 shows the physician shortage area of Shanghai.

Generally speaking, under the fusion method, the physician shortage area covering an area of 56% of Shanghai. Physician-population ratio of these areas ranged from 0.92 to 2.4. What’s more, it’s necessary to pay attention that the poorest area with insufficient health resources where the physician-population ratio is under 1:1000 located primarily in district junction areas. The main reason is that due to restricted resources, government prefer to be responsible for native residents within its jurisdiction. And in Shanghai, majority native residents live in center areas while migrant people mainly settle in the district junction areas. However, with the number of migrant people is increasing in these junction areas, more and more health demand is emerging at the same time. The government have to pay more attention to these areas to guarantee health equality for all residents.

## Conclusion

As shown in the map of Shanghai, areas with physician shortages are located primarily in suburban districts, especially in district junction areas. The result suggests that the government of Shanghai should pay more attention to these areas by investing in new or relocating existing health resources. However, according to the traditional simple supply-demand ratio method, the physician-to-population ratio of Shanghai is 2.5:1000, which already exceeds the standard threshold 2.4:1000 ratio in Shanghai’s regional health planning. For the reason that the health resources distributes unequally, even though the general quantity of health resource is sufficient to residents in an area, it still exist places where are unsufficient with health resources. Therefore, it’s important to find a practicable and valid method to figure out where is unsufficient with health resources. Through this method, valuable suggestions can be put forward and delivered to local health related policy makers and help them take appropriate measures to improve the spatial access to health resources.

In this paper, the physician-to-population ratio, trade area analysis, and the gravity model were used to calculate the accessibility index for third-, second-, and first- class hospitals, respectively. The combined index was then compared with a standard physician-to-population ratio to determine and map the physician shortage areas in Shanghai. Compared to the simple physician-to-population ratio method used by most health department in China, the integrated method used in this paper is demonstrated to be more specific and simultaneously more consistent with the facts, which shows detailed spatial accessibility of health resources. The fusion method is demonstrated to be more accurate and practicable than using a single method to assess spatial access to health resources.

However, we have to recognize that even through the fusion method is better than one single method in accessing spatial access to health resources, there is a long way to go to be a perfect method to assess spatial access to health resources. Note that, in the fusion method, only the number of physicians and residents and the distance between them are considered as the determining factors of spatial health access. Other factors that may also have impact on it are not considered respectively in the fusion model.

## Discussion

This paper showed a method of fusing the proximate method and the gravity method to assess health resource accessibility for Shanghai. Usually, researchers use one method to assess specific health resource accessibility[22–25], because the function and influence of health resources differ by type. However, regardless of which method is used to assess health accessibility, the essence of the problem is a supply to demand ratio. Therefore, in this paper, a population point layer is used as a link to combine all the methods. Using only one method to assess accessibility always leaves unanswered questions. For example, the simple ratio method, the proximal method, and even the 2SFCA method ignore the fact that patients are free to choose health care providers from a large area. Therefore, they are not suitable for assessing access to large-scale hospitals whose patients come from an entire city or even an entire country. The gravity method can help make up for this shortcoming, but it is not practical either because the traffic coefficient *β* suffers from influence by many factors, including traffic conditions, location, and so on. The coefficient is difficult to obtain and changes over time. The goal, then, is to find a way to keep the advantages each method offers while simultaneously removing the disadvantages these methods have. The innovation of this paper is that it is possible to use the most suitable method for each type of health resource and then integrate all the results together using population distribution data. In this way, access to healthcare for an entire city can be displayed in one neat package, letting health policy makers comprehend the distribution of health resources realistically at a macro level.

To create this detailed spatial accessibility visualization for health resources, big data—exact population distribution data, accurate addresses for all health providers, the number of physicians at each provider, the home addresses of inpatients for each hospital—these are fundamental and required by the process. The more accurate the data are, the more realistic the spatial accessibility model will be.

This paper aimed to show a method that could integrate measurements of accessibility of different health resources for Shanghai. Unfortunately, many of the assumptions, additional data, and parameters still need work to improve the results: (1) Both the simple physician-to-population ratio method used at the community level and the proximate area method were applied using the assumption that people residing within a community administrative boundary or within the service area of a regional hospital have the same access to physicians. This assumption is based on the fact that it is difficult for people in a community/service area to seek similar healthcare beyond that community/service area. (2) When using the proximate method to distribute physicians in regional hospitals, the service area was created using the location of regional hospitals and calculating physician-to-population ratio for those service areas. However, doing this means that the method considers distance as the only factor that influences the behavior of patients. People in this model always go to the nearest regional hospital for healthcare. In addition, regional hospitals were not distributed particularly equally in this study, resulting in large gaps between service areas. (3) The travel traffic coefficient *β* used here was estimated by fitting the power model to inpatient probability and the distance between inpatients’ home addresses and the location of hospitals. Actually, the *β* calculated here is the distance decay of the probability of being hospitalized at a certain hospital, and we applied the resulting *β* into the gravity model as the supply decay coefficient of health resources. Furthermore, in the paper, we used Zhongshan Hospital as a typical first-class hospital to estimate *β*; the other 50 first-class hospitals in Shanghai used the same *β*. In ongoing future work, we will do more research to be able to calculate the traffic coefficient β more meticulously. In conclusion, real medical behavior is always complicated because it suffers from influence by so many factors—most of which cannot be revealed easily by quantitative analysis. However, what we can do is put more effort into ensuring that the model used to calculate access to healthcare adheres as closely as possible to reality.
